# Effects of remnant preservation in anterior cruciate ligament reconstruction: A systematic review and meta-analysis

**DOI:** 10.3389/fsurg.2022.952930

**Published:** 2022-09-01

**Authors:** Huanyu Xie, Zicai Fu, Mingjin Zhong, Zhenhan Deng, Chen Wang, Yijia Sun, Weimin Zhu

**Affiliations:** ^1^ Health Science Center, Shenzhen University, Shenzhen, China; ^2^ Department of Sports Medicine, The First Affiliated Hospital of Shenzhen University, Shenzhen Second People's Hospital, Shenzhen, China

**Keywords:** anterior cruciate ligament reconstruction, remnant preservation, meta-analysis, systematic review, anterior cruciate ligament

## Abstract

**Background:**

Compared with standard anterior cruciate ligament (ACL) reconstruction, it is controversial whether anterior cruciate ligament reconstruction (ACLR) with remnant preservation can lead to better clinical outcomes. We conducted a systematic study and meta-analysis to assess the differences in clinical efficacy between the two.

**Method:**

We searched for clinical randomized controlled studies and cohort studies included in the Cochrane library, PubMed, and Embase from March 2012 to March 2022 in English. The included studies were ACLR with or without remant preservation, and the data were extracted and the quality of the included studies was assessed by two authors, respectively. Revman 5.4 was used for statistical analysis and conclusions were presented.

**Result:**

Ten articles containing a total of 777 patients were finally included. There was no significant difference in postoperative Lachman test [OR = 1.66, 95%CI (0.79, 3.49), *P* = 0.18 > 0.05], Tegner score [SMD = −0.13, 95%CI (−0.47, 0.22), *P* = 0.46 > 0.05], synovial coverage rate by second-look arthroscopy [OR = 1.55, 95%CI (0.66, 3.65), *P* = 0.32 > 0.05], the rate of cyclops lesion [OR = 3.92, 95%CI (0.53, 29.29), *P* = 0.18 > 0.05], joint range of motion [SMD = 0.27, 95%CI (−0.13, 0.68), *P* = 0.19 > 0.05] and re-injury rate [OR = 0.57, 95%CI (0.18, 1.74), *P* = 0.32 > 0.05] between the two groups. There were statistically significant differences in postoperative Lysholm score [SMD = 0.98, 95% CI (0.32, 1.64), *P* = 0.004 < 0.05], International Knee Documantation Committee grade (IKDC grade) [OR = 2.19, 95%CI (1.03, 4.65), *P* = 0.04 < 0.05], Pivot shift test [OR = 1.71, 95%CI (1.06, 2.77), *P* = 0.03 < 0.05], KT1000/2000 arthrometer side-to-side difference [SMD = −0.22, 95%CI (−0.42, −0.03), *P* = 0.02 < 0.05], operation time [SMD = 11.69, 95%CI (8.85, 14.54), *P* = 0.00001 < 0.05] and degree of tibial tunnel enlargement [SMD = −0.66, 95%CI (−1.08, −0.23), *P* = 0.002 < 0.05].

**Conclusion:**

This meta-analysis concluded that remnant preservation significantly had better results in terms of patient functional score (Lysholm, IKDC), knee stability (Pivot shift test, postoperative side-to-side anterior laxity) and tibial tunnel enlargement. In terms of complications (incidence of Cyclops lesions, range of motion, re-injury rate), no significant differences were seen between the two groups. Although many studies concluded that remnant preservation could bring better synovial coverage, this meta-analysis indicated that there is insufficient evidence to support it, possibly due to different remnant preservation procedures.The potential risks associated with longer operation times are also worth considering.

## Introduction

The anterior cruciate ligament (ACL) is one of the important structures to maintain the static and dynamic stability of the knee joint. It is located in the joint cavity and surrounded by synovial tissue. ACL injury is one of the most common sports injuries of the knee joint (1). After complete ACL rupture, the broken end of the ACL is gradually encapsulated by synovial tissue, coupled with the special environment in the knee joint, and the injured ligament is usually difficult to heal by itself (2). Anterior cruciate ligament reconstruction (ACLR) has become an effective surgical method for the treatment of ACL injury, which can restore the stability of the knee joint, accelerate the time of return to sport (RTS), and effectively prevent meniscus injury and reduce the risk of arthritis progression (3). However, the postoperative effect did not achieve the desired effect. For example, some patients still have knee instability after surgery, and the re-injury rate and the risk of osteoarthritis still exist (4, 5). In recent years, ACL remnant preservation has become a research hotspot in ACL reconstruction, but the clinical significance, surgical methods and indications of remnant preservation remain controversial.

After ACLR, ACL will go through three biological outcome periods: tissue necrosis, new tissue ingrowth and ligamentization, and then the histological morphology and biomechanical properties of the graft tend to be normal ACL (6). However, in the period of tissue necrosis and new tissue ingrowth, the graft failure load is significantly reduced and it is easy to damage again. A large number of biological and animal experiments have shown that the preservation of ACL remnant can accelerate the synovial coverage of the transplanted ligament, reduce synovial fluid invasion of the transplanted ligament and the inner wall of the bone tunnel, and promote revascularization, ligamentization, and tendon-bone healing (7–10). Beside, residual proprioceptors in the ACL remnant still play a role in stabilizing the knee after ACL injury (11). However, a large number of clinical studies have failed to produce consensus. Some believe that compared with standard reconstruction, ACLR with remnant preservation can bring better clinical efficacy. Some believe that the clinical prognosis of ACLR with remnant preservation is similar to that of standard ACLR, which does not bring better efficacy and may even bring the risk of some complications, such as residual contracture or hyperplasia leading to postoperative knee extension disorder (12).

Several previous reviews also summarized the relevant literature for analysis, but none of them reached a uniform conclusion (12–14). It may be due to insufficient strict literature screening criteria, and no systematic analysis of surgical indications, surgery, etc. Based on previous studies, this study included clinical studies with high grade evidence in the last decade and included all outcome measures available for systematic analysis for systematic studies and meta-analysis whenever possible.

The purpose of this meta-analysis was to compare the clinical outcome of standard ACLR and ACLR with remnant preservation, and provide a reference for clinicians. Our hypothesis is that ACLR with remnant preservation can result in better clinical outcomes.

## Data and methods

### Search strategy and selection criteria

Two authors independently completed a systematic search of three databases (PubMed, EMBASE, Cochrane Library). The base terms used included “anterior cruciate ligament,” “ACL reconstruction,” “remnant,” “preservation,” “remnant-preserved.” This search was limited for studies reporting outcomes in the last 10 years (from March 2012 to March 2022), and was limited to English studies. The included studies (LEVEL OF EVIDENCE: I and II.) were reviewed. According to the inclusion and exclusion criteria, two authors independently selected all articles by reviewing the full text. Any disagreements at the inclusion stage were resolved by discussion with the third author.

### Inclusion and exclusion criteria

The Inclusion criteria for this article are as follows: Randomized controlled trial or cohort study; ACL reconstruction with remnant preservation performed on experimental group, while standard ACL reconstruction without remnant preservation performed on control group, and the surgical techniques were fully described; at least one of the following outcome measures should be reported (postoperative Lysholm score, IKDC score, Tegner score, Lachman test, Pivot-shift test, KT1000/2000 arthrometer side-to-side difference, bone tunnel enlargement, operation time, cyclops lesion, range of motion, re-injury rate, second-look arthroscopic examination); Only human subjects were used. The exclusion criteria are as follows: The full text can't be obtained, the literatures published repeatedly, non-clinical study, retrospective study, including the patients who suffer from postoperative re-injury to reoperation, who are combined with other ligament surgeries, who suffer from fracture combined with open fracture, nerve and blood vessel injury, as well as other knee joint disease history or systemic disease history.

### Data extraction

The study authors, publication time, number of patients, age, gender, time from injury to surgery, postoperative Lysholm score, IKDC score, Tegner score, knee laxity measured by KT1000/2000, bone tunnel enlargement, operation time, re-injury rate and second-look arthroscopic examination were extracted.

### Quality assessment

Data were extracted independently by two arthors, evaluated for quality and reconciled, cross-checked, and in case of discrepancies resolved by discussion or a third investigator decided on their inclusion. Cohort studies used the Newcastle-Ottawa Scale (NOS) score to assess the quality of the literature, and randomized controlled trials were evaluated for the quality of the included studies according to the risk of bias assessment criteria recommended by the Cochrane Handbook for Systematic Reviews.

### Statistical analysis

We used Revman 5.4 for all statistical analyses. Odds ratio (OR or RR) was used for dichotomous data, and weighted mean difference (WMD or SMD) was used for continuous variable data. Both types of indicators were expressed as 95% confidence interval (CI). In terms of heterogeneity test, the studies with good homogeneity (*P* > 0.1 or I^2^ < 50%) used the fixed-effect model for Meta analysis. If there was significant heterogeneity among the studies, the random model was used for Meta analysis. We also performed a subgroup analysis to identify potential differences between remnant preservation with and without tensioning.

## Results

### Literature search

**Figure 1:**
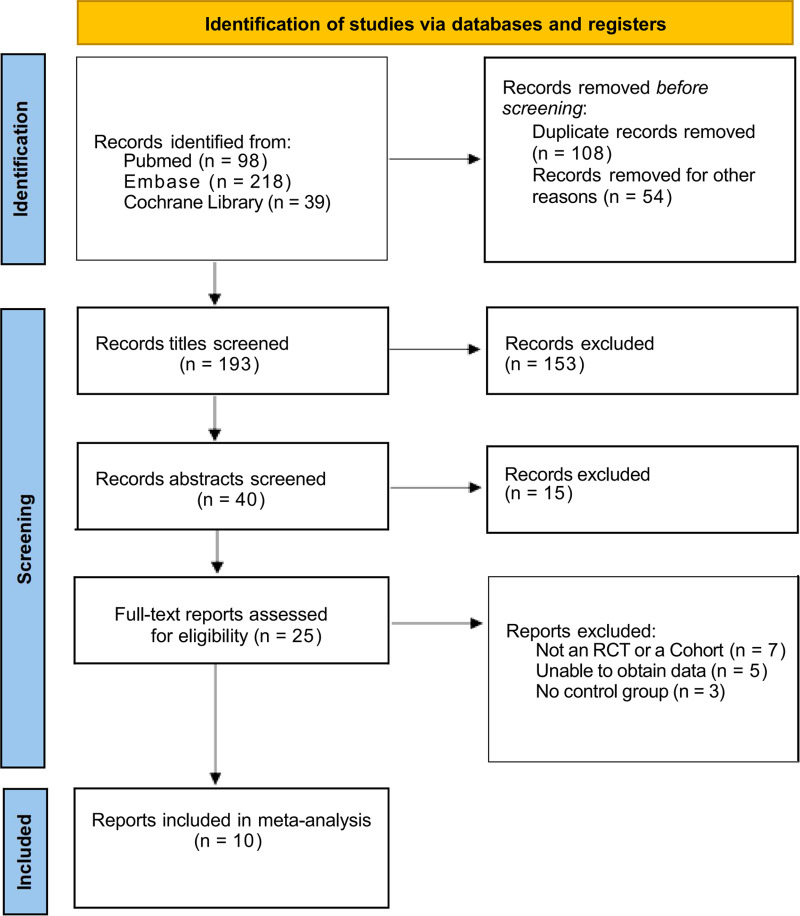
Flow chart of literature screening.

A total of 355 literatures were obtained by searching keywords, including PubMed (*n* = 98), EBSCO (*n* = 218) and Cochrane Library (*n* = 39). After layer-by-layer screening, 10 literatures were finally included (15–24). The literature screening process and results are shown in Figure 1.

Among the 10 included literatures, all were published by English, including 777 patients, 370 cases in the experimental group (ACLR with remnant preservation, ACLR-R) and 407 cases in the control group (standard ACLR, ACLR-S). Table 1 summarized the details of included literatures in this meta-analysis.

**Table 1:** Details of included literatures.

Study	Year	Country	Study type	Sample Size (R/S)	Mean Age (Year, R/S)	Gender M, F (R/S)	Time from injury to surgery, Months (R/S)	Follow-up, Months (R/S)	Type of graft	Surgical technique	Amount of remnant preserved (R)	Outcome	Conclusion	Level of Evidence
Annear	2018	Australia	RCT	20/22	28.1 ± 11.2/29.8 ± 10.0	10,10/13,9	2.6/2.1	40/40	SB/SB, autografts	Group R: preserved tibial remnant Group S: standard ACLR	30–50% of the native ACL	Re-injury rate	No difference in re-injury rate. Higher re-operate rate in Group R	II
Demirağ	2012	Turkey	RCT	20/20	28/31	18,2/18,2	>3 weeks	24.3/24.3	SB/SB, autografts	Group R: single-bundle augmentation technique Group S: standard ACLR	Single remnant bundle preserved	Lysholm score, Lachman test, Pivot-shift test, Tibial tunnel enlargement, Cyclops lesion, Range of motion	No difference in Lysholm scores, Lachman test, Pivot-shift test, and incidences of Cyclops lesions. Less tibial tunnel enlargement in Group R	I
Hong	2012	China	RCT	45/45	34/28	33,12/34,11	10.3 ± 33.7/9.4 ± 25.0	25.8 ± 2.1/25.5 ± 2.4	SB/SB, allografts	Group R: tibial remnant tensioning technique Group S: standard ACLR	>50% of native ACL	IKDC grade, Lachman test, Pivot-shift test, KT1000/2000 arthrometer measurement, Synovial coverage, Operation time	No difference in IKDC grade, Lachman test, Pivot-shift test, KT1000/2000 arthrometer measurement, Synovial coverage, Operation time	II
Kim	2021	Korea	RCT	33/34	33.6 ± 9.5/29.1 ± 7.9	27,6/28,6	1.5 ± 1.7/1.4 ± 1.6	29.2 ± 6.8/28.2 ± 6.2	SB/DB, autografts or allografts	Group R: tibial remnant tensioning technique Group S: standard ACLR	>50% of native ACL (Proximal 1/3 rupture)	Lysholm score, IKDC grade, Lachman test, Pivot-shift test, KT1000/2000 arthrometer measurement, Synovial coverage, Operation time, Cyclops lesion, Range of motion	No difference in Lysholm score, IKDC grade, Lachman test, Pivot-shift test, KT1000/2000 arthrometer measurement, Synovial coverage, Operation time, Cyclops lesion, Range of motion Better graft vascularity in Group R	II
Pujol	2012	France	RCT	29/25	31.2/28.6	16,13/17,8	5.3/4.1	12/12	SB/SB, autografts	Group R: single-bundle augmentation technique Group S: standard ACLR	PL remnant bundle preserved	IKDC grade, Pivot-shift test	No difference in IKDC grade, Pivot-shift test	I
Zhang	2014	China	RCT	27/24	23.5 ± 4.2/25.3 ± 6.1	19,4/21,5	12.7 ± 11.6/10.2 ± 9.0	24.4/25.2	SB/SB, autografts	Group R: preserved tibial remnant Group S: standard ACLR	Not available	Lysholm score, KT1000/2000 arthrometer measurement, Tibial tunnel enlargement	No difference in Lysholm scores, KT1000/2000 arthrometer measurement Less tibial tunnel enlargement in Group R	I
Kim	2017	Korea	Cohort	25/25	28.6 /26.5	21,4/22,3	1.8/1.5	26.8/28.9	SB, allografts /SB, autografts	Group R: tibial remnant tensioning Group S: standard ACLR	Not available	Lysholm score, Tegner score, KT1000/2000 arthrometer measurement, Synovial coverage, Operation time, Cyclops lesion	No difference in Lysholm score, Tegner score, KT1000/2000 arthrometer measurement, Synovial coverage, Cyclops lesion More Operation time in Group R	II
Kondo	2015	Japan	Cohort	81/98	29 ± 13/30 ± 14	44,37/54,44	7 ± 16/12 ± 21	24/24	DB/DB, autografts	Group R: preserved tibial remnant Group S: standard ACLR	Not available	Lysholm score, IKDC grade, Pivot-shift test, KT1000/2000 arthrometer measurement, Cyclops lesion	No difference in Lysholm score, IKDC grade, Cyclops lesion Better knee stability (Pivot-shift test, KT1000/2000 arthrometer measurement) in Group R	II
Masuda	2018	Japan	Cohort	40/39	30 ± 13/29 ± 14	18,22/20,19	4 ± 3/18 ± 35	12/12	DB/DB, autografts	Group R: preserved tibial remnant Group S: standard ACLR	Not available	Lysholm score, IKDC grade, Tegner score, Pivot-shift test, KT1000/2000 arthrometer measurement,	No difference in Lysholm score, IKDC grade, Tegner score, KT1000/2000 arthrometer measurement Less femoral AM tunnel enlargement in Group R	II
Nakayama	2017	Japan	Cohort	50/75	26.6 ± 9.7/26.4 ± 11.7	28,22/40,35	Not available	12/12	DB/DB, autografts	Group R: tibial remnant tensioning technique Group S: standard ACLR	>50% of native ACL	Lysholm score, KT1000/2000 arthrometer measurement, Cyclops lesion, Re-injury rate	No difference in Lysholm score, KT1000/2000 arthrometer measurement, Re-injury rate More incidence of Cyclops lesion in Group R Better synovial coverage and graft integrity in Group R	II

RCT, randomized controlled trial; Cohort, Cohort study; M, male; F, female; SB, single-bundle; DB, double-bundle; Group R, ACLR with remnant preservation; Group S, standard ACLR.

**Figure 2:**
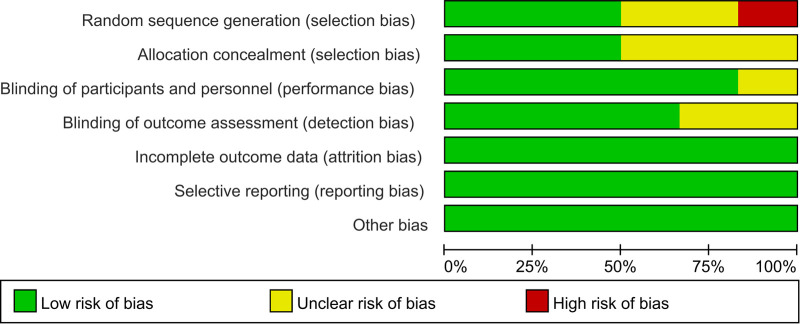
Quality evaluation results of included literature.

**Figure 3:**
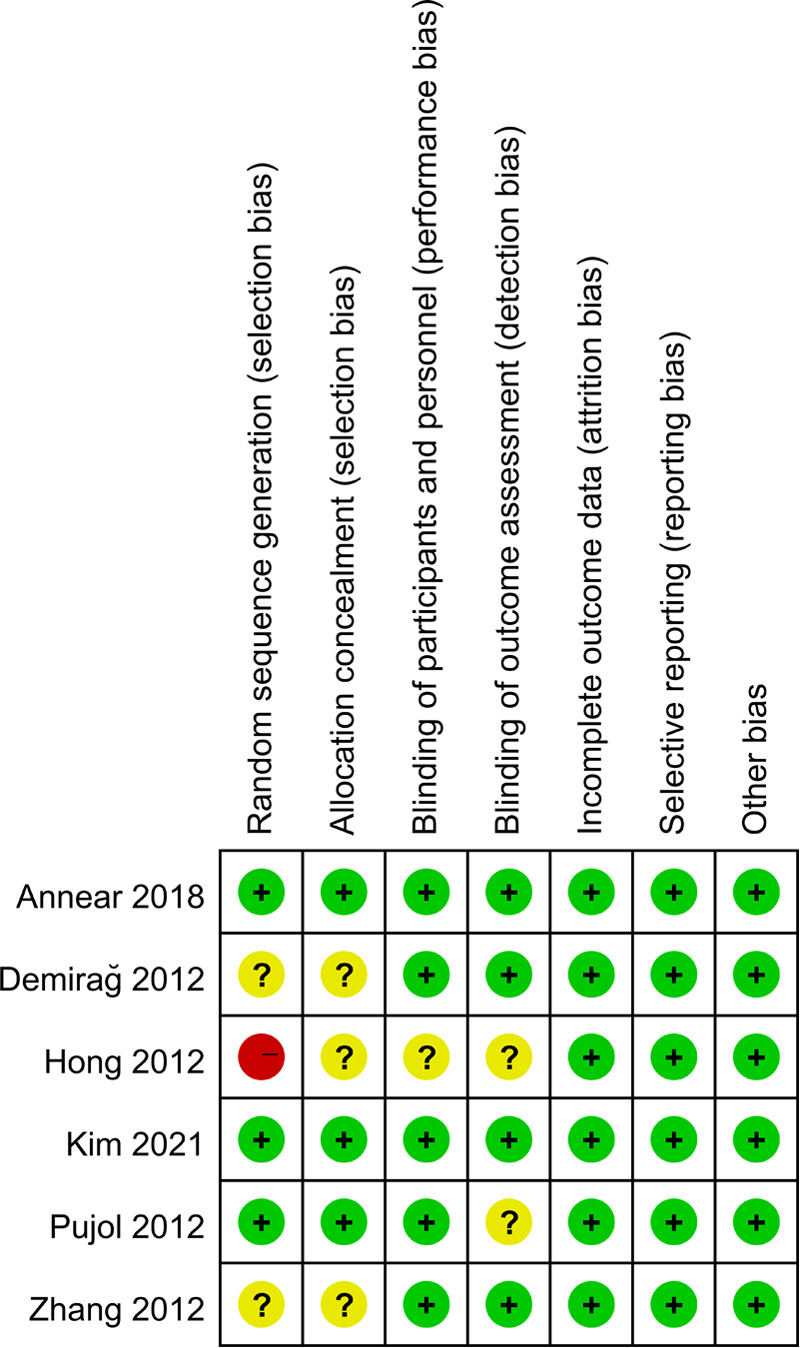
Summary of literature quality assessment results of included studies.

6 included articles were randomized controlled trials (15–20), and 4 were cohort studies (21–24). One of the four cohort studies scored 8 (22), one study scored 7 (23), and two scored 6 (21, 24). Randomized controlled trials evaluated the quality of included studies according to the risk of bias assessment criteria recommended by the Cochrane Handbook for Systematic Reviews, as shown in Figure 2, 3.

### Patient subjective score

#### Lysholm score

**Figure 4:**
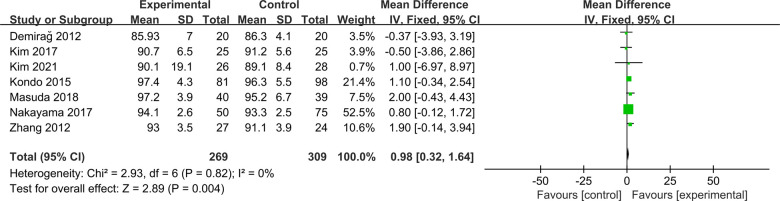
Forest pot for Lysholm score.

Seven studies were included to compare postoperative Lysholm scores between ACLR-R and ACLR-S (16, 18, 20–24). The fixed-effect model was selected for analysis based on the heterogeneity test results (*P* = 0.82, *I*^2^ = 0%). The postoperative Lysholm score in the ACLR-R group was better than that in the ACLR-S group, and the difference was statistically significant [SMD = 0.98, 95% CI (0.32, 1.64), *P* = 0.004 < 0.05], as shown in Figure 4.

#### IKDC grade

**Figure 5:**
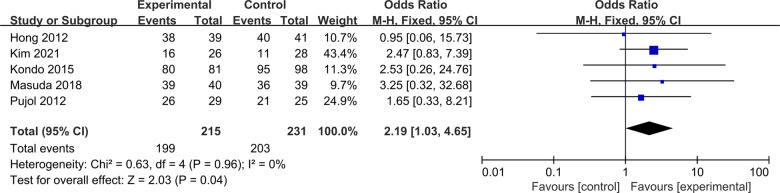
Forest pot for IKDC grade.

Five studies were included to compare postoperative IKDC grade between ACLR-R and ACLR-S (17–19, 22, 23). The fixed-effect model was selected for analysis based on the heterogeneity test results (*P* = 0.96, *I*^2^ = 0%). The postoperative IKDC grade A/B probability in the ACLR-R group was better than that in the ACLR-S group, and the difference was statistically significant [OR = 2.19, 95%CI (1.03, 4.65), *P* = 0.04 < 0.05], as shown in Figure 5.

#### Tegner score

**Figure 6:**
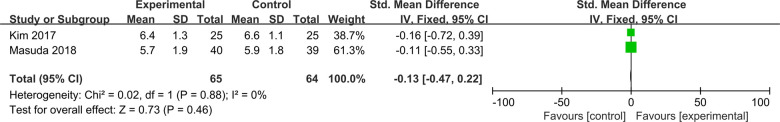
Forest pot for Tegner score.

Two studies were included to compare postoperative Tegner score between ACLR-R and ACLR-S (21, 23). The fixed-effect model was selected for analysis based on the heterogeneity test results (*P* = 0.88, *I*^2^ = 0%). There was no significant difference in postoperative Tegner scores between the two groups [SMD = −0.13, 95%CI (−0.47, 0.22), *P* = 0.46 > 0.05], as shown in Figure 6.

### Knee stability

#### Lachman test

**Figure 7:**
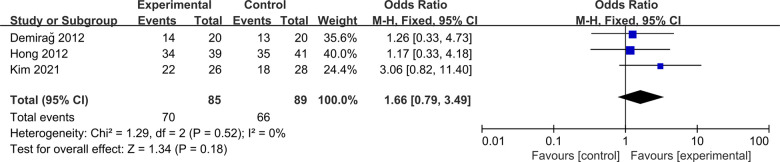
Forest pot for Lachman test.

Three studies were included to compare postoperative Lachman test between ACLR-R and ACLR-S (16–18). The fixed-effect model was selected for analysis based on the heterogeneity test results (*P* = 0.52, *I*^2^ = 0%). There was no significant difference in postoperative Lachman test between the two groups [OR = 1.66, 95%CI (0.79, 3.49), *P* = 0.18 > 0.05], as shown in Figure 7.

#### Pivot-shift test

**Figure 8:**
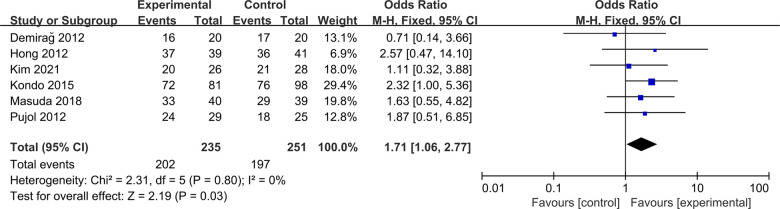
Forest pot for Pivot-shift test.

Six studies were included to compare postoperative Pivot-shift test between ACLR-R and ACLR-S (16–19, 22, 23). The fixed-effect model was selected for analysis based on the heterogeneity test results (*P* = 0.80, *I*^2^ = 0%). The negative rate of postoperative Pivot shift test in ACLR-R group was more than that in ACLR-S group, and the difference was statistically significant [OR = 1.71, 95%CI (1.06, 2.77), *P* = 0.03 < 0.05], as shown in Figure 8.

#### KT1000/2000 arthrometer measurement

**Figure 9:**
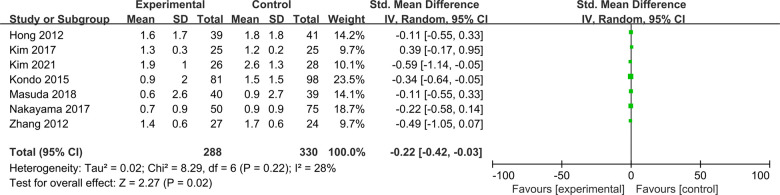
Forest pot for KT1000/2000 arthrometer measurement.

Seven studies were included to compare postoperative side-to-side anterior laxity measured by KT1000/2000 arthrometer between ACLR-R and ACLR-S (17, 18, 20–24). The fixed-effect model was selected for analysis based on the heterogeneity test results (*P* = 0.22, *I*^2^ = 28%). The postoperative side-to-side anterior laxity measured by KT1000/2000 arthrometer in the ACLR-R group was less than that in the ACLR-S group, and the difference was statistically significant [SMD = −0.22, 95%CI (−0.42, −0.03), *P* = 0.02 < 0.05], as shown in Figure 9.

### Synovial coverage

**Figure 10:**
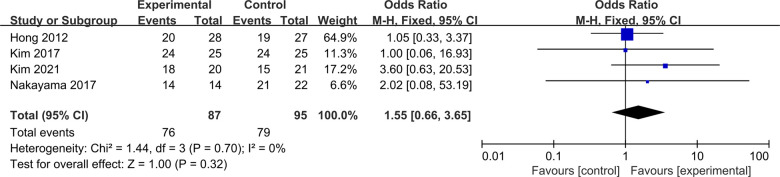
Forest pot for synovial coverage.

Four studies were included to compare postoperative synovial coverage between ACLR-R and ACLR-S (17, 18, 21, 24). Four studies were included to compare postoperative synovial coverage between ACLR-R and ACLR-S. The fixed-effect model was selected for analysis based on the heterogeneity test results (*P* = 0.70, *I*^2^ = 0%). The probability of patients with postoperative synovial coverage >50% was similar between the two groups, and the difference was not statistically significant [OR = 1.55, 95%CI (0.66, 3.65), *P* = 0.32 > 0.05], as shown in Figure 10.

### Operation time

**Figure 11:**
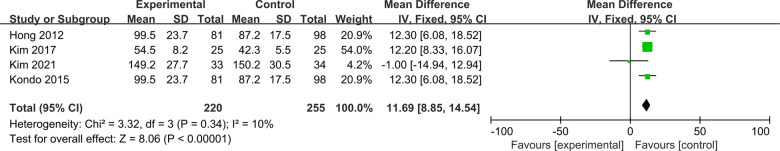
Forest pot for operation time.

Four studies were included to compare operation time between ACLR-R and ACLR-S (17, 18, 21, 22). The fixed-effect model was selected for analysis based on the heterogeneity test results (*P* = 0.34, *I*^2^ = 10%). The operation time in the ACLR-R group was more than that in the ACLR-S group, and the difference was statistically significant [SMD = 11.69, 95%CI (8.85, 14.54), *P* = 0.00001 < 0.05], as shown in Figure 11.

### Complications

#### Tibial tunnel enlargement

**Figure 12:**
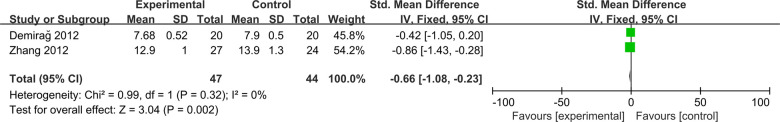
Forest pot for tibial tunnel enlargement.

Two studies were included to compare tibial tunnel enlargement between ACLR-R and ACLR-S (16, 20). The fixed-effect model was selected for analysis based on the heterogeneity test results (*P* = 0.32, *I*^2^ = 0%). The tibial tunnel enlargement in the ACLR-R group was less than that in the ACLR-S group, and the difference was statistically significant [SMD = −0.66, 95%CI (−1.08, −0.23), *P* = 0.002 < 0.05], as shown in Figure 12.

#### Cyclops lesion

**Figure 13:**
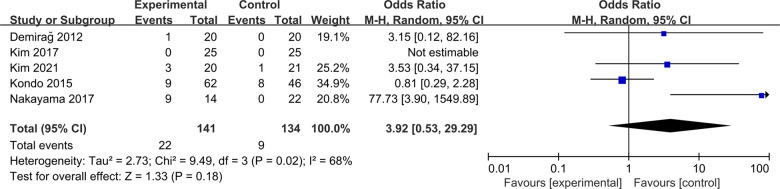
Forest pot for Cyclops lesion.

Five studies were included to compare cyclops lesion between ACLR-R and ACLR-S (16, 18, 21, 22, 24). The Random-effect model was selected for analysis based on the heterogeneity test results (*P* = 0.02, *I*^2^ = 68%). The cyclops lesion was similar between the two groups, and the difference was not statistically significant [OR = 3.92, 95%CI (0.53, 29.29), *P* = 0.18 > 0.05], as shown in Figure 13.

#### Range of motion

**Figure 14:**
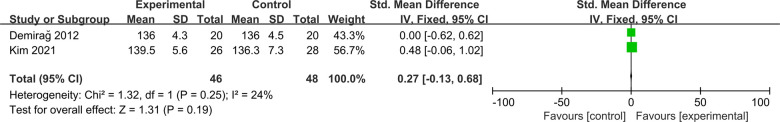
Forest pot for range of motion.

Two studies were included to compare postoperative range of motion (ROM) between ACLR-R and ACLR-S (16, 18). The fixed-effect model was selected for analysis based on the heterogeneity test results (*P* = 0.25, *I*^2^ = 24%). The postoperative ROM was similar between the two groups, and the difference was not statistically significant [SMD = 0.27, 95%CI (−0.13, 0.68), *P* = 0.19 > 0.05], as shown in Figure 14.

#### Re-injury rate

**Figure 15:**
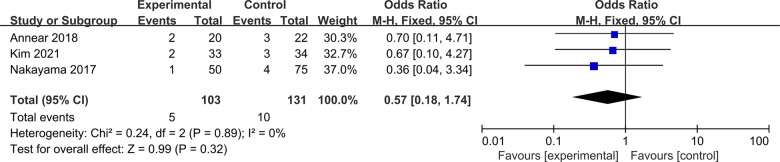
Forest pot for re-injury rate.

Three studies were included to compare re-injury rate between ACLR-R and ACLR-S (15, 18, 24). The fixed-effect model was selected for analysis based on the heterogeneity test results (*P* = 0.89, *I*^2^ = 0%). The rate of re-injury was similar between the two groups, and the difference was not statistically significant [OR = 0.57, 95%CI (0.18, 1.74), *P* = 0.32 > 0.05], as shown in Figure 15.

### Subgroup analysis

Subgroup analysis was performed according to technique of remnant preservation (remnant preservation with and without remnant tensioning), as shown in Table 2. In the subgroup of Non-remnant tensioning, significant differences were found in Lysholm score, Pivot-shift test and side-to-side difference between ACLR-P and ACLR-S. In the subgroup of remnant tensioning, we could see high heterogeneity of postoperative side-to-side difference between ACLR-P and ACLR-S due to the study of Kim (21). In this trial, the ACLR-P used allograft tendons, while the ACLR-S used autologous tendons. Exclusion of this trial altered the result of the side-to-side difference [SMD = −0.26, 95%CI (−0.51, −0.02), *P* = 0.04 < 0.05] (*P* = 0.39 and *I*^2^ = 0% for heterogeneity) between ACLR-P and ACLR-S.

**Table 2:** Results of subgroup analysis.

	Remnant tensioning				Non-remnant tensioning			
	MD/RR	95% CI	Heterogeneity (*P*/*I*^2^)	*P* value	MD/RR	95% CI	Heterogeneity (*P*/I^2^)	*P* value
Lysholm score	MD = 0.71	[−0.16, 1.59]	0.76/0%	0.11	MD = 1.49	[0.43, 2.54]	0.74/0%	0.006
IKDC grade	OR = 2.17	[0.79, 5.99]	0.53/0%	0.13	OR = 2.86	[0.56, 14.50]	0.88/0%	0.20
Pivot-shift test	OR = 1.24	[0.53, 2.86]	0.55/0%	0.62	OR = 2.04	[1.09, 3.95]	0.61/0%	0.03
Side-to-side anterior laxity	SMD = −0.15	[−0.48, 0.19]	0.1/52%	0.40	SMD = −0.27	[−0.52, −0.02]	0.40/0%	0.03

IKDC, international knee documentation committee.

## Discussion

Our results suggest that compared with standard ACLR, ACLR with remnant preservation had better results in terms of Lysholm scores, IKDC grade, Pivot shift test, postoperative side-to-side anterior laxity, operation time and degree of tibial tunnel enlargement. However, there was no significant difference between the two groups in terms of Lachman test, Tegner score, synovial coverage rate, complications (incidence of Cyclops lesions, range of motion), and re-injury rate. These results suggest that the ACLR with remnant preservation can promote graft healing, increase knee stability, prevent tibial tunnel widening, and have similar or even better clinical outcomes than standard ACLR.

Knee stability includes both static and dynamic stability. Static stability mainly refers to the mechanical traction of ligaments, while dynamic stability refers to the perception and control of the knee during movement. ACL is not only a static stability device for the knee joint, but also has a role in maintaining the dynamic stability of the knee joint because of proprioceptors (25). Patients with ACL injury will have knee instability, swelling and pain. Without timely treatment, patients will have secondary meniscus and articular cartilage injury, and even the risk of progression of arthritis will be increased, which will seriously affect the function of the patient's knee joint. It can be seen that restoring knee stability is the most important therapeutic aim of ACL reconstruction. After ACLR, both graft ligament healing and proprioceptive recovery affect knee stability.

After ACLR, the graft ligament is incompetent and undergoes four stages: ischemia, necrosis, proliferation, and ligamentization in the joint cavity, followed by a tendency to normal ACL (26). Synovialization and vascularization of the graft are important stages of the biological healing process. Good synovial coverage can promote the reconstruction of blood supply of the ACL graft and bring better knee function and stability. Synovialization and vascularization of the graft are important stages of the biological healing process. Good synovial coverage can promote the reconstruction of blood supply of the ACL graft and bring better knee function and stability (27). Animal studies had showed that after ACL complete injury, preserving the ACL remnant, which has a vascular-rich synovium, a large number of fibroblasts, myofibroblasts, and vascular-derived stem cells, can promote the ability of vascular regeneration by promoting the expression of vasoactive factors around the graft, and eventually promote synovialization and ligamentization of the graft (28, 29). Many follow-up studies had also demonstrated that preserving the remnant results in better synovial coverage, and that good synovial coverage may contribute to knee stability (30, 31). However, our study concluded that there is no significant difference between the two groups in terms of the probability of patients with postoperative synovial coverage > 50%. This result may be due to the differences in surgical procedures of remnant preservation and ACLR among the four included articles. In the study by Nakayama et al, both groups performed double-bundle ACLR using autograft (24). In the study by Kim et al, both groups used single-bundle ACLR, while the ACLR-P group used allograft and ACLR-S group used autograft (21). Kim et al performed single-bundle ACLR for ACLR-P, and double-bundle for ACLR-S group, and both of the groups used allograft or autograft (18). Hong et al performed single-bundle ACLR using allograft (17). Only Nakayama et al placed the remnant between the AM and PL bundle grafts (24), the others passed the graft through the center of the tibial remnant (17, 18, 21). So we supposed that our result due to the following reasons: (1) Synovialization of allograft is worse than autograft, (2) The graft passes through the center of the tibial remnant, which means more contact area with the remnant, resulting in the remnant wrapping the graft tendon and sealing of the tibial tunnel adequately (32). At the same time, not only these four articles, but all the included literature do not have a uniform standard for the quality of remnant preservation. Kim et al found that only preserving the remnants fully covered with synovium can have better synovial coverage at the second microscopy than non-remnant preservation (33). It meant that a remnant with poor synovial coverage cannot contribute to postoperative synovial coverage. Meanwhile, the small sample size is also a factor that affects the result.

Besides the second-look arthroscopic examination, MRI is also commonly used to evaluate ligamentization of the graft. Signal/noise quatient (SNQ) is a common indicator for assessing the degree of graft ligamentization after ACLR. Lower SNQ represents lower graft water content, more ligamentization, and better biomechanical properties (34). Remnant preservation is an independent associated factors of graft SNQ (35). Takahashi et al. performed a retrospective analysis and showed that SNQ values of ACL grafted tendon at 2 years after surgery in ACLR-R Group were better than those in ACLR-S Group (30). In our Meta analysis, only one study evaluated the SNQ value, and the results showed that the mean SNQ values were compared and showed no signifificant intergroup differences at 1 year (18). In this study, they performed remnant-tensioning single-bundle and double-bundle ACLR, and . But when they used DCE-MRI to assess graft vascularity, the results showed that the ACL-R group had a richer graft vascularity than the ACLR-S group.

In addition to static stability, dynamic stability is also particularly important. ACL is also a proprioceptive organ, included a large number of proprioceptors, which are mainly distributed near the femoral and tibial insertion of the ligament and are particularly important for maintaining the dynamic stability of the knee joint (36). During knee flexion, extension, rotation and other movements, ACL receives the corresponding mechanical traction. The proprioceptor receives the signal and generates nerve impulses, which are transmitted to the central nervous system, forming reflexes and proprioception. Then muscles adjacent to the joints contract to complete the role of protecting and controlling the knee joint (37). After ACL injury, mechanoreceptors and conduction pathways are injured, resulting in affected neuromuscular reflexes and ultimately, and affecting knee proprioception and stability (38). Studies have shown that after ACL injury, proprioceptors still exist at ACL remnant, which are involved in completing part of the proprioceptive function, and the number of receptors is positively correlated with the proprioceptive level of the knee joint (39, 40). Animal experiments showed that the number and density of proprioceptors were significantly higher after ACLR-R, comparing to standard ACLR (41). Angle reproduction and angle thresholds are commonly used clinically to assess patient proprioception. Two literatures were included in this study to evaluate the patient's postoperative proprioception, and both concluded that remnant preservation was beneficial to the recovery of proprioception. However, due to the differences in the measurement method and data recording method in detail, it is impossible to make a comprehensive analysis.

Knee stability was primarily assessed by physical examination (Lachman test and Pivot shift test) and KT1000/KT2000 arthrometer. Anteroposterior knee stability was assessed by the Lachman test and KT1000/KT2000 arthrometer, while rotational stability was assessed by the pivot shift test. Eight articles were included in this meta-analysis to assess knee stability, with three assess the Lachman test, six assess the pivot shift test, and seven assess the side-to-side anterior laxity by KT1000/KT2000 arthrometer. In this meta-analysis, it was concluded that remnant preservation could improve knee stability, but there was no significant change in postoperative subjective function scores. Some scholars have previously proposed that there is no relationship between objective measurement results and patients' subjective feelings, but objective measurements are superior in assessing patient knee stability (42).

Bone tunnel enlargement is one of the important indicators affecting the prognosis of ACLR. After ACLR, the bone is absorbed or dissolved under the combined stimulation of biological and mechanical factors (inflammatory factors, immune response, bone quality, bone tunnel position, graft fixation method, graft material, etc.), resulting in tibial tunnel enlargement. From a physical point of view, the tibial remnant preservation can seal the graft, separate the bone tunnel and joint cavity and reduce synovial fluid penetration into the bone tunnel (43). Tight wrapping of the graft by the remnant tissue reduces micromotion of the graft in the bone tunnel. From a biological healing point of view, the blood supply of the remnant can help the graft to revascularization and crawl instead, promotes the biological healing between the graft and the bone tunnel, and also reduces the micromotion between the graft and the bone tunnel (41). our meta-analysis shows a similar conclusion that the tibial tunnel enlargement in ACLR-R group was significantly lower than that in ACLR-S group. In addition, some scholars have proposed that poor bone tunnel positioning is also one of the reasons affecting bone tunnel enlargement. In the past, it was believed that preserving the remnant tissue could affect the localization of the bone tunnel. With improvements in surgical techniques, several studies have demonstrated that remnant does not affect bone tunnel positioning.

Cyclops lesions refers to the formation of a fibrovascular tissue nodule in the front of ACL graft. most of which are asymptomatic (44, 45). Cyclops syndrome is an important cause of reoperation after ACLR due to symptomatic extension dysfunction caused by cyclops impingement in the intercondylar fossa, with an overall incidence of about 2%–47% (22). Some scholars believe that preservation of ACL remnant increases the incidence of cyclops lesions after ACL reconstruction. However, the pathogenesis of cyclops lesions produced by ACL remnant is inconclusive and may be due to the development of fibers or inflammatory hyperplasia due to remnant stimulation. Also, there is no study could clearly demonstrate the association between remnant preservation and cyclops syndrome. Recent studies have shown that remnant preservation does not lead to an increased incidence of cyclops lesions, and even if it produces intercondylar notch hyperplasia, it does not affect the patient's postoperative clinical manifestations. A cohort study suggests that remnant preservation is not associated with symptomatic cyclops lesions, possibly because hypervascular scar tissue may also be generated after removal of the remnant (46). Removal of the remnant can cause increased bleeding, which can lead to scar tissue, and eventual cyclops lesions. But remnant preservation does not debride a large amount of remnant tissue, so reduced bleeding. It has even been shown that cyclops lesions do not lead to early postoperative extension dysfunction, but extension dysfunction will promote the proliferation of intercondylar notch nodules and ultimately form cyclops lesions. The amount of remnant preserved also had no effect on the generation of cyclops lesions. The results of this study also shows similar conclusions, at the second-look arthroscopy, remnant preservation did not cause an increase in cyclops lesions, and there was no significant difference in postoperative range of motion.

Four literatures analyzed the operation time and our analysis showed that the operation time was significantly longer in group A than in group B. Only one study showed no significant difference in operative time between the two groups, probably due to the fact that group ACLR-R performed remnant-tensioning single-bundle ACLR while group underwent double-bundle ACLR (18). The increase in surgery time may put the knee at increased risk of infection. Besides, the longer operation time means that the use of tourniquets is longer, and it is worth considering whether there is an impact on the recovery of muscle strength of the quadriceps muscle postoperatively.

Remnant preservation with tenision is believed to promote biological healing of the graft, as well as bring better preservation of mechanoreceptors due to residual mechanoreceptors receiving constant mechanical stimulation (47). Also, tenisioning the tibial remnant can avoid impingement, because it can prevent the loose injury ACL from curling up on the tibial footprint (47). Depending on whether tension was applied to the tibial remnant, we performed a subgroup analysis. The results showed that preservation without remnant tensioning had significant advantages in terms of Lysholm score, IKDC grade, Pivot-shift test and side-to-side difference, but group preservation with remnant tensioning does not show the significant superiority, comparing to Group ACLR-S. The results may be due to the points mentioned above, the differences in ACLR procedures, remnant placement, and amount and quality of remnant preservation (33, 40, 48, 49).

This study has some limitations. First, previous studies have shown that ACLR-R can preserve proprioceptions in the ACL remnant, and in this study, only two of the included articles underwent proprioception assessment, but we were not able to perform analyses and comparisons because of the inconsistent measurement method and data processing method. 2, The follow-up time of the studies included in this study was less than 3 years. 3, The technique of remnant preservation of the included articles was not uniform.

Despite these limitations, this study included eleven articles with a high level of evidence, and all were RCT and cohort studies, in the past 10 years. Although the techniques of remnant preservation are not uniform, this study is the first to provide the subgroup analysis of surgical techniques.

## Conclusion

This meta-analysis concluded that remnant preservation significantly had better results in terms of patient functional score (Lysholm, IKDC), knee stability (Pivot shift test, postoperative side-to-side anterior laxity) and tibial tunnel enlargement. In terms of complications (incidence of Cyclops lesions, range of motion, re-injury rate), no significant differences were seen between the two groups. Although many studies concluded that remnant preservation could bring better synovial coverage, this meta-analysis indicated that there is insufficient evidence to support it, possibly due to different remnant preservation procedures.The potential risks associated with longer operation times are also worth considering.

## Data Availability

The original contributions presented in the study are included in the article/Supplementary Material, further inquiries can be directed to the corresponding author/s.
